# Semaglutide-Associated Gastric Pneumatosis

**DOI:** 10.14309/crj.0000000000001579

**Published:** 2024-12-27

**Authors:** Andrew M. Turunen, Reilly A. Coombs, Sushil Kumar Garg

**Affiliations:** 1 School of Medicine, Medical College of Wisconsin-Central Wisconsin, Wausau, WI; 2 Division of Gastroenterology, Mayo Clinic Health System, Eau Claire, WI

**Keywords:** semaglutide, gastric pneumatosis, GLP-1 agonist

## Abstract

Semaglutide, a glucagon-like peptide-1 receptor agonist, used for Type 2 diabetes mellitus and more recently for weight loss, often causes gastrointestinal adverse effects such as delayed gastric emptying and abdominal discomfort. Current literature has not described an associated case of gastric pneumatosis with semaglutide use. We report a 61-year-old man on semaglutide for 9 months with gastric pneumatosis. Symptoms resolved on discontinuation. Clinicians should be vigilant for significant gastrointestinal adverse effects, including pneumatosis with semaglutide use.

## INTRODUCTION

Semaglutide, a glucagon-like peptide-1 receptor agonist, modulates insulin and glucagon secretion, delaying gastric emptying. Initially, designed for the treatment of Type 2 diabetes mellitus (T2DM), its recent popularity stems from its weight loss potential. Literature highlights common side effects such as nausea, vomiting, diarrhea, hypoglycemia, and gastroesophageal reflux disease, with rare but serious risks including thyroid C-cell tumors and pancreatitis.^1^ Its prolonged half-life, compared with liraglutide, accentuates gastrointestinal (GI) motility slowdown.^2^ A recent study by Liu et al observed a higher incidence of GI adverse effects (AEs) with semaglutide, when compared with its glucagon-like peptide-1 receptor agonist peers.^2^ As semaglutide usage continues to surge post-US Food and Drug Administration approval in 2017 and with the obesity crisis in America, ongoing vigilance for adverse effects remains crucial. This case illustrates a 61-year-old man with T2DM on semaglutide presenting with gastric pneumatosis and portal venous gas in perigastric venules and liver.

## CASE REPORT

A 61-year-old man with a complex medical history including gastroesophageal reflux disease, irritable bowel syndrome, sleep apnea, T2DM, dyslipidemia, generalized anxiety disorder, and bipolar type 2 disorder struggled to maintain his blood sugar levels. The patient had a hemoglobin A1c of 10 on his daily medications of as follows: metformin XR 2,000 mg, glimepiride 8 mg, glargine 50 units, lispro 8 units with meals, and dulaglutide (1.5 mg/0.5 mL). To better control his blood sugar, the patient was transitioned to semaglutide (2 mg/1.5 mL) subcutaneous weekly injections. After 3 months of semaglutide use, there was minimal improvement and the dose of semaglutide was increased to 4 mg/3 mL injections. Subsequently, the patient experienced severe nausea, diarrhea, and headaches, attributed to the increased semaglutide dose, leading to a reduction back to 2 mg/1.5 mL injections weekly.

**Figure 1.**
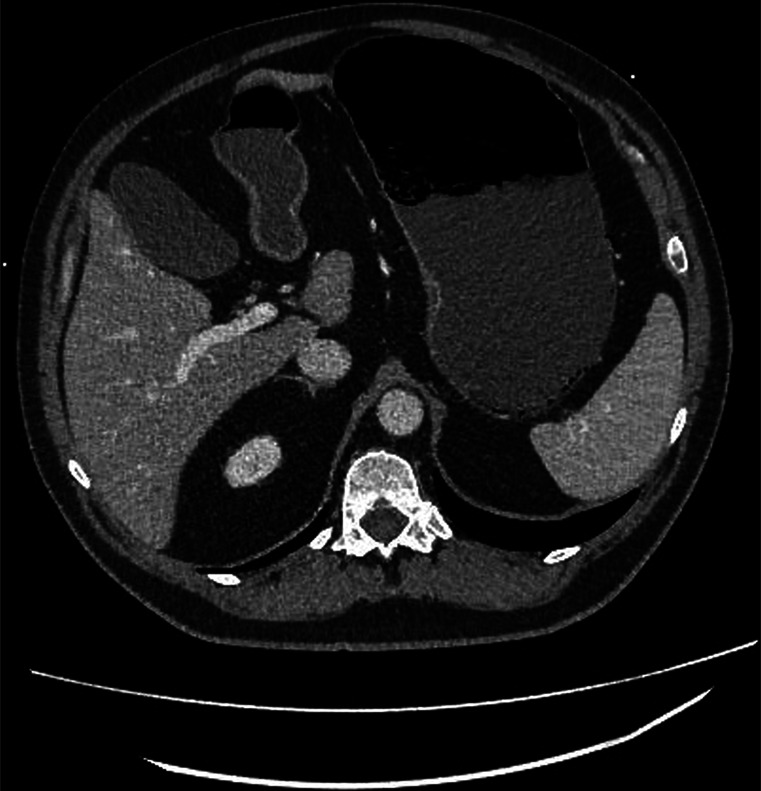
Computed tomography of the abdomen showing gastric pneumatosis in the gastric cardia and proximal fundus.

**Figure 2.**
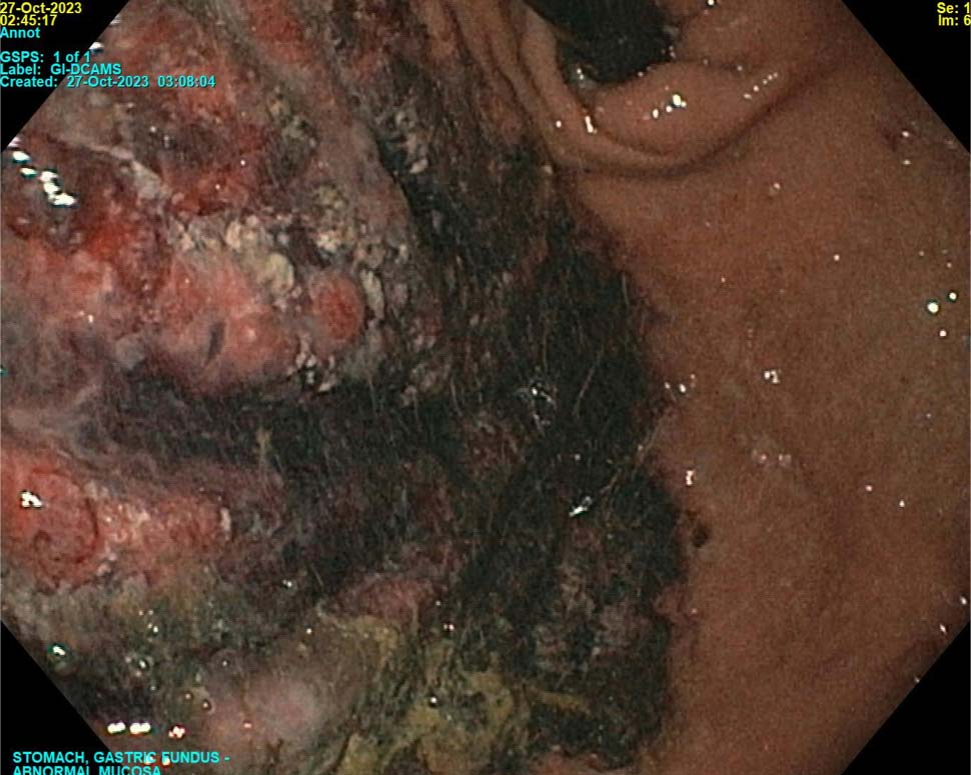
Esophagogastroduodenoscopy findings showing congested, hemorrhagic, and petechial mucosa in the gastric fundus and body.

Nine months after semaglutide dose reduction, the patient presented to the emergency department (ED) with abdominal pain, nausea, and vomiting. The patients vitals on arrival to the ED were as follows: temperature 96.6°F, pulse 50, heart rate 86, respiratory rate 19, blood pressure 106/56, and SpO2 of 99% on room air. Symptoms began abruptly, with diarrhea on day 1, progressing to worsening malaise and persistent diarrhea on day 2, and multiple episodes of vomiting on day 3 (the day he arrived to the ED). In the ED, the patient denied any recent travel, sick contacts, or flatulence. Physical examination revealed mild, diffuse abdominal discomfort without distention or guarding. A computed tomography (CT) of the abdomen and pelvis with intravenous contrast revealed possible gastric wall ischemia with dusky appearance of the gastric cardia and proximal fundus, along with pneumatosis and portal venous gas (Figure 1). Esophagogastroduodenoscopy revealed congested, hemorrhagic, and petechial mucosa in the gastric fundus and body, with retained gastric fluid and a normal duodenum (Figure 2). Pathology demonstrated superficial lamina propria hemorrhage and mucosal erosion in the gastric mucosa.

On arrival at the ED, in addition to the CT and esophagogastroduodenoscopy, laboratory investigations were negative for coronavirus disease 2019 and influenza. Hematological analysis indicated leukocytosis (10.5 × 10^9^/L) with a left shift, along with elevated lactate (3.0 mmol/L) and C-reactive protein (15 mg/L). In addition, there was low magnesium level (1.6 mg/dL). Metabolic panel findings included a slightly reduced bicarbonate level (20 mmol/L) and elevated glucose (201 mg/dL). Blood cultures were drawn to rule out infectious causes and were negative after 5 days. The patient denied chronic nonsteroidal anti-inflammatory drug use, tobacco use, or a history of alcohol consumption, helping us to rule out other potential contributors to the gastric pneumatosis/ischemia. The profound nausea and vomiting secondary to the use of semaglutide was identified as a potential contributing factor to this patient's gastric pneumatosis. There was no indication of vasculitis on the abdominal CT with intravenous contrast, as none of the typical findings were observed. Specifically, there was no arterial wall thickening, perivascular fat stranding, arterial stenosis, occlusion, aneurysm formation, or contrast enhancement of the vessel walls. In addition, there was no evidence of celiac artery or superior mesenteric artery stenosis and thrombosis.

The patient was admitted for further evaluation and management. Hospital treatment involved twice-daily proton-pump inhibitors, intravenous antibiotics, one day of nasogastric decompression, and a gradual transition to a blenderized diet. Metformin and semaglutide were temporarily held, and sliding scale insulin was initiated for glycemic control. After 4 days of hospitalization, the patient was discharged in stable condition, with all medications reinstated except for semaglutide, which was discontinued as the suspected culprit for this event. Pantoprazole (4 mg twice daily) and the remaining antibiotic course were continued postdischarge.

At the 1-week hospital follow-up, the patient reported feeling well, with no nausea, vomiting, or significant abdominal pain while on the blenderized diet. He denied experiencing fever, chills, melena, or hematochezia. A follow-up endoscopy was conducted one month after discharge, revealing no abnormalities in the esophageal, gastric, or duodenal mucosa. Pathological examination confirmed the normalcy of the antral and fundic mucosa and ruled out *Helicobacter pylori* infection.

## DISCUSSION

Given semaglutide's recent introduction to the market, the off-target effects of semaglutide are still being evaluated in real-world populations. A 2020 study evaluated the efficacy and safety of semaglutide vs sitagliptin and found that of those taking 0.5 mg of semaglutide, 20.2% experienced diarrhea, 7.7% nausea, and 4.9% vomiting as adverse side effects.^3^ It has also been suggested that, for both oral and subcutaneous semaglutide, higher doses are often more frequently with GI adverse effects.^4^ Wharton et al found that once weekly semaglutide in adults was more associated with GI adverse effects than placebo.^5^

Gastric pneumatosis is the presence of air within the stomach wall and is considered an uncommon occurrence. The major mechanisms of gastric pneumatosis include gastric ischemia, intramural gastric infections, gastric mucosal disruptions (ex. forceful vomiting), or due to extension of supradiaphragmatic air (such as secondary to pneumomediastinum).^6^ In our case study, the patient exhibited common GI AEs, including diarrhea, nausea, and vomiting, alongside signs of gastric ischemia and pneumatosis. CT imaging also revealed portal venous gas in perigastric venules and liver. Given this patient's extensive history of uncontrolled diabetes, it is also possible that he may have had diabetic gastroparesis. The addition of semaglutide, which carries a known side effect of exacerbating gastroparesis, could have contributed to gastric ischemia by increasing gastric pressure and subsequently reducing gastric blood flow. In animal studies, increased gastric pressure has been shown to decrease gastric blood flow.^7^ Another potential explanation for the development of gastric pneumatosis in this patient could the result of profound nausea and vomiting which could have caused mucosal disruption. The combination of mucosal disruption from vomiting along with gastric ischemia from increased gastric pressure could have caused these findings. Regardless of the exact mechanism, on cessation of semaglutide, the patient's symptoms ameliorated, suggesting a potential causal relationship.

To date, no documented instances of semaglutide-induced gastric pneumatosis exist. As semaglutide gains traction for its weight loss efficacy, it is imperative to thoroughly evaluate its AEs when assessing its therapeutic benefits. While commonplace AEs such as diarrhea, nausea, and vomiting are acknowledged, it is crucial to recognize the potential for more severe GI complications such as gastric pneumonitis and ischemia. A comprehensive understanding and vigilance regarding both common and rare GI side effects are paramount in clinical decision making surrounding semaglutide utilization.

## DISCLOSURES

Author contributions: A. Turunen: Performed a thorough literature review, compiled patient case information, and drafted the report and assisted in finalizing it. R. Coombs: Contributed significantly to editing the manuscript and assisting in finalizing it. SK Garg: Guarantor of article and provided oversight, guidance, and significant feedback and edits of the final manuscript.

Financial disclosure: None to report.

Previous presentation: Case report was presented as a poster presentation at the American College of Physicians—Wisconsin Chapter 2024 Annual Scientific Meeting; on September 7, 2024; Wisconsin Dells, WI.

Informed consent was obtained for this case report.

## References

[1] Ozempic Ozempic.: Dosing, contraindications, side effects, and pill pictures - epocrates online. (n.d.). https://www.epocrates.com/online/drugs/7978/ozempic

[2] Liu L Chen J Wang L Chen C Chen L. Association between different GLP-1 receptor agonists and gastrointestinal adverse reactions: A real-world disproportionality study based on FDA adverse event reporting system database. Front Endocrinol (Lausanne). 2022;13:1043789. 36568085 10.3389/fendo.2022.1043789 PMC9770009

[3] Ji L Dong X Li Y Efficacy and safety of once-weekly semaglutide versus once-daily sitagliptin as add-on to metformin in patients with type 2 diabetes in SUSTAIN China: A 30-week, double-blind, phase 3a, randomized trial. Diabetes Obes Metab. 2021;23(2):404–14. 33074557 10.1111/dom.14232 PMC7839591

[4] Smits MM Van Raalte DH. Safety of semaglutide. Front Endocrinol (Lausanne). 2021;12:645563. 34305810 10.3389/fendo.2021.645563 PMC8294388

[5] Wharton S Calanna S Davies M Gastrointestinal tolerability of once-weekly semaglutide 2.4 mg in adults with overweight or obesity, and the relationship between gastrointestinal adverse events and weight loss. Diabetes Obes Metab. 2022;24(1):94–105. 34514682 10.1111/dom.14551 PMC9293236

[6] Schattner A Glick Y. Gastric pneumatosis and its varied pathogenesis. QJM. 2020;113(10):747–8. 32240308 10.1093/qjmed/hcaa108

[7] Varhaug JE Svanes K Lysen LJ Holm P. The effect of intragastric pressure on gastric blood flow after partial devascularization of the stomach in cats. Eur Surg Res. 1980;12(6):415–27. 7262131 10.1159/000128149

